# Relative frequency and risk factors for long-term opioid therapy following surgery and trauma among adults: a systematic review protocol

**DOI:** 10.1186/s13643-018-0760-3

**Published:** 2018-07-18

**Authors:** M. Gabrielle Pagé, Irina Kudrina, Hervé Tchala Vignon Zomahoun, Daniela Ziegler, Pierre Beaulieu, Céline Charbonneau, Jennifer Cogan, Raoul Daoust, Marc O. Martel, Andrée Néron, Philippe Richebé, Hance Clarke

**Affiliations:** 1 Centre de recherche du Centre hospitalier de l’Université de Montréal (CRCHUM), Tour Saint-Antoine S01-112, 850 rue St-Denis, Montreal, QC H2X 0A9 Canada; 2 Department of Anesthesiology and Pain Medicine, Faculty of Medicine, Université de Montréal, Pavillon Roger-Gaudry, local S-712, C.P. 6128, Succ. Centre-ville, Montréal, QC H3C 3J7 Canada; 3 Department of Family Medicine, Faculty of Medicine, McGill University, 5858 Ch de la Côte des Neiges, Montreal, QC H3S 1Z1 Canada; 4 Department of Social and Preventive Medicine, Faculty of Medicine, Université Laval, Pavillon Ferdinand-Vandry, 1050 ave de la Médecine, local 2431, Québec, QC G1V 0A6 Canada; 5 Health and Social Services Systems, Knowledge Translation and Implementation Component of the Quebec SPOR-SUPPORT Unit, Pavillon Landry-Poulin, 2525, Chemin de la Canardiere, Quebec, QC G1J 0A4 Canada; 6 Department of Information Science, Hotel Dieu, 3840 Saint-Urbain Pavillon Olier 4e étage porte 2-428, Montreal, QC H2W 1T8 Canada; 7 Anesthesiology Department, Centre hospitalier de l’Université de Montréal, 1051 rue Sanguinet, Montreal, QC H2X 0C1 Canada; 8 Association Québécoise de la douleur chronique (AQDC), bureau 403, 2030 boul. Pie-IX, Montreal, QC H1V 2C8 Canada; 9 Department of Anesthesiology, Montreal Heart Institute, 5000 Bélanger, Montreal, QC H1T 1C8 Canada; 10 Emergency medicine, Hôpital du Sacré-Coeur de Montréal, 5400 Boul. Gouin Ouest, Montreal, QC H4J 1C5 Canada; 11 Faculty of Dentistry, McGill University, 2001 Avenue McGill College, |500, Montreal, QC H3A 1G1 Canada; 12 Clinique de la douleur, Département de pharmacie, Centre hospitalier de l’Université de Montréal, 5e, Pav C – C0550, 1051 rue Sanguinet, Montreal, QC H2X 0C1 Canada; 13 Department of Anesthesiology, Hôpital Maisonneuve-Rosemont, 5415 Assumption Blvd, Montreal, QC H1T 2M4 Canada; 14 Department of Anesthesia and Pain Management, Toronto General Hospital, University Health Network, 200 Elizabeth St 3EN-464, Toronto, ON M5G 2C4 Canada; 15 Transitional Pain Service, Toronto General Hospital, University Health Network, 200 Elizabeth St 3EN-464, Toronto, ON M5G 2C4 Canada

**Keywords:** Systematic review protocol, Surgery, Trauma, Opioids, Risk factors, Prolonged therapy, Long-term use

## Abstract

**Background:**

When patients have been on opioid therapy for more than 90 days, more than half of them continue using opioids years later. Knowing that long-term opioid consumption could lead to harmful side effects including misuse, abuse, and addiction, it is important to understand the risks of transitioning to prolonged opioid therapy to reduce its occurrence. Perioperative and trauma contexts are ideal models commonly used to study such transition. Long-term use of opioids might be associated with transformation of acute pain to chronic, which might be an example of a risk factor. The objectives of this knowledge synthesis are to examine the relative frequency and the risk factors for transitioning to long-term opioid therapy among patients who have undergone a surgical procedure or experienced a trauma.

**Methods:**

The proposed study methodology is based on Preferred ReportIng Systematic Reviews and Meta-Analysis Protocols (PRISMA-P) statements on the conduct of systematic review and meta-analysis, the MOOSE Guidelines for Meta-Analyses and Systematic Reviews of Observational Studies, and the *Cochrane Handbook for Systematic Review of Interventions*. A systematic literature search will include multiple databases: Cochrane Central, EMBASE, MEDLINE, PsycINFO, CINHAL, PubMed, and the grey literature. We will identify studies related to opioid use beyond acute/subacute pain control after surgery or trauma. Two of the reviewers will screen all retrieved articles for eligibility and data extraction then critically appraise all identified studies. We will compile a narrative synthesis of all results and conduct a meta-analysis when feasible. As available data permits, we will perform a subgroup analysis of vulnerable populations.

**Discussion:**

This systematic review will contribute to the prevention and harm reduction strategies associated with prescription opioids by identifying risk factors leading to the unwarranted long-term opioid therapy. The identification of common risk factors for long-term opioid therapy will help to orient further research on pain management as well as offer key therapeutic targets for the development of strategies to prevent prolonged opioid use.

**Systematic review registration:**

This protocol was registered in PROSPERO on March 2, 2018; registration number CRD42012018089907.

## Introduction

Opioid prescriptions have significantly increased over the last two to three decades, especially in the USA and across Canada [1, 2]. Opioids remain among the most widely used analgesic medications in the treatment of chronic non-cancer pain (CNCP). Post-surgical pain management is a great example of nearly systematic use of opioids as 80% of surgical patients are prescribed opioids [3]. Indeed, untreated postoperative pain could have detrimental consequences such as development of CNCP, deterioration of quality of life and poorer medical outcomes [4]. It is not rare that opioid therapy is initiated without a clear treatment plan, and prescriptions are renewed when patients complain of persistent pain thus leading unintentionally to prolonged opioid therapy [5]. This practice has numerous societal implications since the storage of unused medications [6, 7], improper discarding practices of excess narcotic analgesics [8] and/or used medications (e.g., fentanyl patches), and deficient inter-professional communication (e.g., family doctors and specialists) [9] could pose a public health threat. This may be complicated by various non-coordinated trials of opioids by different providers, some of which may exceed recovery time, in addition to the insufficient patient follow-up and elevated caseload of many clinicians.

In this context, long-term opioid therapy refers to the use of prescription opioids that were initially intended to treat an acute pain condition and continued beyond the recovery phase [10], typically for more than 90 days.

The perioperative model offers an ideal context to study the transition from acute to chronic pain [11] (pain that lasts for longer than expected, typically more than 3–6 months [12]) and acute to long-term opioid therapy [13, 14]. When the onset of pain and the date of initiation of opioid therapy are known, baseline measurements can be collected perioperatively to examine predisposing factors. The initial time-limited utilization becomes prolonged for many patients; 27% of CNCP patients on opioid therapy (months to years following their first prescription) were initially prescribed opioids following surgery [15]. Recent guidelines on the management of postoperative pain [16] recognize the need for the round-the-clock analgesia over the first 24 h postoperatively and often longer for major surgeries. The guidelines advocate for specific actions to facilitate hospital discharge of patients on opioid therapy but recognize that there is insufficient knowledge of how to perform a postoperative opioid taper [16]. This lack of knowledge most likely contributes to the unplanned development of prolonged opioid therapy. Considering that 2.5 million surgeries are performed yearly in Canada [17], thousands of these patients are potentially at risk for transition to prolonged and long-term opioid therapy.

Similar to the perioperative model, trauma is often associated with high intensity acute pain, and more than half of trauma patients are discharged from the hospital with an opioid prescription [10, 18]. Incidence of long-term opioid therapy following trauma may be high [19–22]. For example, 39% of workers who filed a compensation claim and who received an opioid prescription remained on opioid therapy 1 year later [23]. Among patients with fractures, more than 40% and 30% continue taking prescribed opioids 6 and 12 months post-fracture, respectively [19, 24].

Understanding how an initially time-limited opioid prescription turns into a long-term therapy is essential to optimize treatment and minimize potential harm associated with opioid therapy. To date, we have found only one narrative review on opioid consumption after surgery [25] that briefly discusses the incidence and risk factors for long-term opioid therapy after surgery and emphasizes several strategies to facilitate opioid cessation. However, the authors omitted several relevant studies from this review [26–28]. There was no critical appraisal of the literature or attempt to integrate results across the studies. This work does not examine the long-term opioid therapy in other contexts such as trauma. Lastly, because the scientific search method is not reported, the review is subject to significant influence from the writers’ opinions [29].

There is a dearth of literature supporting effectiveness of long-term opioid therapy [30] and its potential negative consequences [31, 32]. As such, strategies promoting opioid-related harm reduction must target risk factors for transitioning to long-term opioid therapy. This can only be based on a strong understanding of how opioid therapy becomes prolonged in the first place. A systematic review of the relative frequency of and the risk factors for long-term opioid therapy among populations for which opioids are frequently used (e.g., 80% of surgical patients [3]) would generate such understanding. Due to the heterogeneity in the literature, it has been difficult to come to a specific conclusion. There is no consensus on what constitutes the most common risk and protective factors for the long-term use of opioids. The incidence of prolonged opioid use in different populations remains unknown [25]. Knowledge of what constitutes modifiable risk factors for unnecessary long-term opioid therapy is of vital importance to the prevention of secondary opioid harm. This systematic review seeks to contribute to the knowledge of harm prevention; therefore, we present here details of the systematic review protocol.

## Goals of systematic review

The two main objectives of this systematic review are to:

1. Examine the relative frequency of prolonged and chronic opioid therapy

and

(2) Identify the risk factors for transitioning to prolonged and chronic opioid therapy among adult patients who have experienced physical trauma requiring hospitalization and/or undergone surgery of any type within a hospital setting and who received an opioid prescription at discharge or within the 2 weeks following hospital discharge.

Data will be examined separately for opioid-naive patients and patients on opioid therapy prior to surgery/trauma. If data permit, the relative frequency and risk factors will be examined in distinct populations (e.g., women, elderly, young adults, indigenous people, and individuals suffering from mental illness).

## Methods

**Fig. 1:**
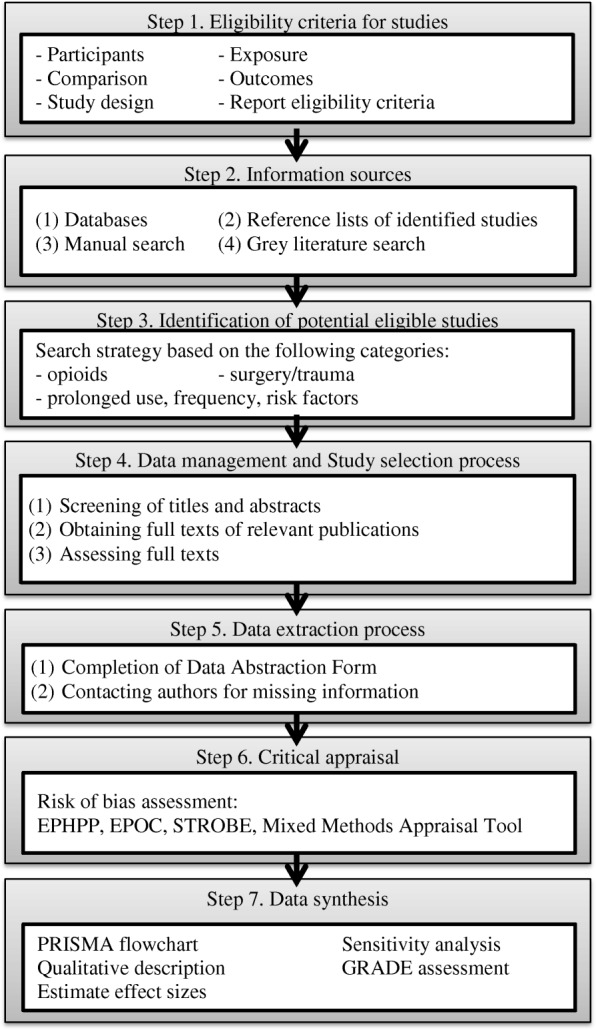
Steps of systematic review

This protocol and its proposed methodology are based on Preferred ReportIng Systematic Reviews and Meta-Analysis Protocols (PRISMA-P) statement [33] on the conduct of systematic review and meta-analysis, the MOOSE Guidelines for Meta-Analyses and Systematic Reviews of Observational Studies [34], and the *Cochrane Handbook for Systematic Review of Interventions* [35]. The systematic review is composed of seven steps (see Fig. 1).

### Operationalization of key constructs

- Surgery refers to any minor or major operative or manual procedure involving instrumental manipulation of the living human tissues (performed by a surgeon [36] or a non-surgical specialty). Only surgeries typically performed in-hospital (day surgery or inpatient surgery) will be included.
- Trauma refers to an injury to the living tissue (e.g., body wound) that is caused by an external agent (blunt force or penetrating) [37] or other physical stressors (e.g., falls, burns, and fractures) which may or may not require any further surgical intervention. Purely psychological traumas without a physical component will not be considered for inclusion.
- Opioids refer to a class of drugs targeting opioid receptors. Consistent with other systematic reviews, all opioid agonists/partial agonists will be considered, administered using pure or mixed formulations, and all systemic routes of administration will be included (routes of administration can change over time).
- Opioid therapy duration and frequency: categorization of opioid consumption will be based on a temporal definition (as opposed to dose-related). We will report the frequency of use as a number of prescriptions per considered period.
  - *○ Transient* will be defined as at least one opioid prescription (prescribed, distributed, or reported) of any length within the first 44 days post discharge and at least one prescription of opioids between 1.5 and < 3 months (45 to 89 days) post hospital discharge from the surgery/trauma.
  - *○ Prolonged opioid therapy* will be defined as at least one prescription (prescribed, distributed, or reported) within the first 44 days post discharge, at least one prescription of opioids between 1.5 and 3 months post hospital discharge and at least one prescription of opioids of any length between 3 and 6 months post hospital discharge from the surgery/trauma, irrespective of the dose.
  - *○ Chronic opioid therapy* will be defined as at least one opioid prescription (prescribed, distributed or reported) of any length more than 6 months post hospital discharge following surgery/trauma in addition to meeting criteria for prolonged opioid therapy.

These definitions are based on time-to-cessation [38] of opioids following surgery. Long-term opioid therapy is used in this article to encompass both prolonged and chronic opioid use (a generic term describing opioid consumption that exceeds 90 days following hospital discharge post-event).

- Daily dosing: there is no consensus on definitions of lower versus high-dose therapy [39]. Even morphine equivalence daily doses (MEDD) between 20 and 50 mg are associated with increased risk of overdose and death [40]. When data are available, we will convert reported daily doses for long-term opioid therapy into their respective MEDD. Daily opioid consumption will be reported as follows:
  - *○ Low dose* defined as a total daily dose less than 50 MEDD
  - *○ Moderate dose* defined as a total daily dose between 50 and 90 MEDD
  - *○ High dose* defined as a total daily dose above 90 MEDD
- Type of opioid use will be defined as episodic or continuous
  - *○ Episodic use* is opioid consumption > 3 months with total days of opioid supply < 120, and total number of prescriptions filled < 10
  - *○ Continuous use* is opioid consumption > 3 months with ≥120 days of opioid supply and ≥ 10 total number of prescriptions filled [5, 41, 42].
- Preoperative and pre-trauma opioid use status: patients will be classified as preoperative or pre-trauma opioid users if they have received opioid supplies more than one month before surgery or trauma. Patients who received opioids only in the days before surgery (30 days or less) will be considered as opioid naive given that opioids are frequently prescribed to patients before surgery for postoperative pain control.

### Eligibility criteria

Eligibility criteria are defined based on the PICOS (Participants, Intervention/Exposition, Control, Outcomes, and Study design) approach [43]. Study selection will be based on the criteria outlined below.

#### Participants

##### Inclusion criteria

Studies will be included if they meet the following criteria: adults (≥ 18 years old), surgery (minor/major performed at the hospital) and/or physical trauma/injury (requiring hospitalization) and requiring opioid-based analgesia of any duration post-hospital discharge (at least one opioid prescription filled at discharge or within 2 weeks of hospital discharge). Consistent with other reviews, in case of mixed populations (e.g., a study that has included some cancer patients within their sample) for which data cannot be obtained by authors, studies with ≥ 75% of patients meeting the inclusion criteria will be accepted [41]. Purely psychological traumas without a physical component will not be considered for inclusion. Methadone and buprenorphine are almost never first-line analgesic agents; however, they will be included if time for initiation and analgesic purpose are clearly identified in a study. Since the principles of pediatric analgesia rely on differing therapeutic criteria [44, 45], only adults are included in this review.

##### Exclusion criteria

Studies will be excluded if any of the following conditions apply: study participants suffering from cancer pain, requiring end-of-life care, with addiction substitution/maintenance therapy, with lack of consideration for presence/absence of opioid therapy pre-surgery/trauma, and having undergone another surgical procedure or experienced another trauma during the study follow-up period. Studies in which a time frame for opioid initiation and treatment duration cannot be identified will be excluded. In cases where studies examine only the 3-month outcomes with no mention of opioid intake between discharge and follow-up, these studies will be included if information is available to determine that the opioid prescription at 3 months is taken because of the indexed event. Given that opioid-naive patients and patients on opioid therapy before surgery or trauma will be examined separately, studies with mixed populations that do not allow for subgroup analyses and the examination of incidence and risk factors separately (e.g., for opioid-naive patients and preoperative/pre-trauma opioid users) will be excluded.

#### Exposure

Exposure will consist of having received at least one opioid prescription within 2 weeks following hospital discharge post-event.

#### Comparison

Comparison, when applicable, would consist of comparing the presence/absence or degree of risk factors between patients who developed long-term opioid therapy vs. those who discontinued opioids in the acute phase.

#### Outcomes

##### Objective 1

Frequency of opioid use, daily dosing, and type of opioid use at different time points (45 days, 90 days, 6 months, and 12 months following hospital discharge post event) will be reported for each type of surgery and trauma. There must be an evidence of current opioid use (i.e., opioid prescription filled within that time window) at the pre-determined cut-off points for transient, prolonged, and chronic opioid therapy and no period greater than 90 days without opioid consumption at any point since hospital discharge [46]. This information will have been sourced from the medico-administrative databases or medical charts, or patient self-report data.

##### Objective 2

In the identification of biopsychosocial risk and protective factors for long-term opioid therapy post-discharge, three types of risk factors will be assessed related to (1) patients, (2) health care providers, and (3) health care system. Data sources for patient factors will include validated self-report questionnaires as well as medical charts/medico-administrative databases. Data sources for health care providers and health care system factors will include medical charts, medico-administrative databases, and clinical/health care systems.

#### Study design

##### Inclusion

The following study designs will be included: experimental designs (randomized controlled trial, quasi-experimental designs), observational studies (cross-sectional, cohort, case-control, case series), and mixed methods studies.

##### Exclusion

We will exclude editorials and commentaries. Studies with a follow-up period shorter than 1.5 months following hospital discharge post-event will be excluded.

#### Limits

*Language*: Only articles published in English or French will be considered.

*Publication status and publication year*: Articles published since 1998 or in press will be considered for inclusion. This cut-off was chosen given that it follows the first consensus statement by the American Academy of Pain Medicine and the American Pain Society on the use of opioids in the treatment of chronic non-cancer pain [47] leading to significant practice change in the use of opioids for CNCP management.

### Information sources

The following four literature categories will be searched:

1. Databases: The following databases will be searched by a professional study librarian (DZ) and reviewed by two independent librarians: MEDLINE (1998–), PubMed (1998–), CINAHL (1998–), PsycINFO (1998–), EMB Review (1998–), EMBASE (1998-), Cochrane Database of Systematic Reviews, and Cochrane Central Register of Controlled Trials.
2. Reference lists of identified studies and any relevant review articles found will be screened;
3. Additional manual search of relevant journals particularly for trauma given the variety of key words used for indexing (Trauma and Acute Care, Annals of Emergency Medicine, Emergency Medicine International, European Journal of Trauma & Emergency Surgery);
4. Grey literature (Google scholar, Pro Quest Dissertation and Theses; published reports).

The search bibliography will also be circulated to all study authors who have expertise in the domain.

A focused search for distinct populations (elderly, women, young adults, indigenous people, and individuals suffering from mental illness) will also be performed using Google Scholar. To conduct this specific search, we will add key terms (e.g., Indigenous) to the search strategy in Google Scholar and compare the articles retrieved to those found in the original search. We will continue until 10 pages with the maximum number of articles have been reviewed or until saturation point has been reached (when Google Scholar does not identify any articles not previously included), whichever happens first.

### Search strategy

The search strategy was elaborated by our information expert (librarian) (DZ) and our field experts (GP, IK) and reviewed by a second independent librarian. We have reviewed key words, MeSH terms, and search strategies used in relevant original studies and literature reviews. We plan to conduct the search for the relevant studies based on the following terms and derived terminology from the four pre-identified conceptual groups: (1) opioid (including synonyms, generic, and brand names of medications); (2) surgery or trauma (including names of specific conditions typically not indexed under these terms, e.g., knee arthroplasty, fracture, and burn); (3) transient/prolonged/long-term/frequency/prevalence/incidence or risk factors. The complete search strategy for MEDLINE database is presented in the Appendix. These search terms will be adapted for each of the databases to be considered. For each of the databases, controlled vocabulary (MeSH, EMTREE, and others), and free-text searching will be used. A manual search of the bibliographies of each of the original studies and relevant reviews included will be conducted by GP and research assistants to identify other potential relevant references.

### Study records

#### Data management

All search results will be merged using the EndNote software, and duplicate records will be removed. The study selection process will be performed using the software *DistillerSR* (DistillerSR, Evidence Partners, Ottawa, Canada). Titles and abstracts will be independently screened for eligibility by two reviewers. Reviewers will include GP, IK, and experienced research assistants. The full text of potentially relevant reports will be further analyzed for eligibility. Disagreements will be resolved by consensus and if needed, by requesting the opinion of the third reviewer. To ensure consistency across reviewers, a review of selection process will be performed after 10% of the identified articles have been reviewed. Levels of inter-rater agreement [48] (kappa statistics) will be documented.

#### Selection process

A research assistant will obtain the full text of all relevant publications and further analyze the information against the defined eligibility criteria. Two reviewers will be assigned to each publication, and input from the third reviewer will be solicited in case of disagreement. The following information will be recorded in this modified Effective Practice and Organization of Care (EPOC) form: surname of first author, year of first report, date form completed, names of reviewers extracting data, report title, type of publication, funding source, conflicts of interests, and study characteristics (study type, participant, intervention types, and outcomes).

A manual search using authors’ names will be conducted to identify single studies that have been published more than once. The review authors will not be blinded to journal titles and study authors of institutions.

#### Data extraction and reporting

For the data extraction of selected studies, we will adapt the Cochrane *EPOC Data Abstraction Form and Data Extraction Instructions* [49]). Data extraction will be done within the DistillerSR software. For the specific needs of this systematic review, GP and the research assistant will pilot the adapted EPOC form with instructions on 10 randomly chosen studies to refine and finalize it.

Data extraction will be independently done by GP and research assistants using the adapted recording form. The data extraction form sections are designed to extract information concerning all aspects of each study, including population and study characteristics, methods used to measure exposition and outcome characteristics, results on association measures of interest, and results applicability. Authors will be contacted as needed to obtain missing information.

Patient data will be organized into the broad categories of risk factors similar to those for chronic postoperative pain [11], namely sociodemographic, psychosocial, and medical/surgical/trauma characteristics. Health care professional risk factors will be categorized into sociodemographic, psychosocial, and professional (e.g., experience, type of profession, and prescribing practices). Lastly, health care system risk factors will be categorized into infrastructure (e.g., proximity of pharmacies and access to health care professional) and economic (e.g., percentage of reimbursement of opioid prescription costs).

### Data items and outcomes

#### Objective 1: relative frequency of opioid therapy

##### Primary outcome

The primary outcome will be the relative frequency of long-term opioid therapy (≥ 90 days) following hospital discharge post trauma and/or post surgery. This will be reported as a proportion of patients on opioid therapy out of the total number of eligible patients (refer to the section “Operationalization of key constructs” for more details).

##### Secondary outcomes

Secondary outcomes will examine the relative frequency of opioid therapy at different stages following hospital discharge (transient, prolonged, and chronic use periods). Relative frequencies will be examined based on preoperative/pre-trauma opioid status and if data permits based on minor vs. major surgeries [50].

#### Objective 2: risk and protective factors for long-term opioid therapy

Three types of risk factors will be assessed (patients, health care providers, and health care system).

##### Primary outcome

Identification of biopsychosocial risk and protective factors for long-term opioid therapy post-discharge (≥ 90 days).

##### Secondary outcomes

In identification of biopsychosocial risk and protective factors at the different stages following hospital discharge, some literature suggests that the risk factors for the development (3-month status) vs. maintenance (12-month status) of postoperative pain are not the same (e.g., [51]). As such, as data permits, this review will examine risk factors for prolonged opioid use separately from chronic opioid use, the latter allowing for risk factors measured 3 months after hospital discharge to be examined.

### Risk of bias in individual studies

Two from the five reviewers (GP, IK, research assistants) will review each included article separately to assess the risk of bias for each outcome of interest. In case of disagreement, a discussion will take place to achieve consensus; otherwise, a third reviewer will appraise the study. Different assessment tools will be used depending on each study design: the Effective Public Health Practice Project (EPHPP) Quality Assessment Tool for Quantitative Studies for cohort studies and case-control studies [52], the EPOC Risk of Bias Tool for RCT [53], the Quality Assessment Tool for Observational Cohort and Cross-Sectional Studies [54], and Mixed Methods Appraisal Tool for Systematic Mixed Studies Reviews [55]. Eight categories (selection, study design, confounders, blinding, data collection methods, withdrawals/drop-outs, integrity of delivered intervention, analyses) will be assessed using 21 items. The EPHPP tool has a good inter- and intra-rater reliability [52, 56] and is one of the top tools for critical appraisal in systematic reviews [57]. Source of study funding (e.g., sponsored by pharmaceutical companies or not) will also be examined.

### Synthesis

#### Data synthesis process

First, results of the study selection process will be described using a PRISMA Flowchart [58], and the statistic of inter-rater agreement (kappa) [48] will be reported. Second, we will perform a qualitative description of the population, the studies included, the risk factors identified, and the outcome characteristics using simple frequency counts and a narrative approach. Third, only the studies in which data on risk factors and the outcomes of interest are available to estimate the relative frequency and effect size will be included in the meta-analysis.

#### Data synthesis reporting

If effect sizes are available or calculable in two or more studies for a specific outcome, a meta-analysis will be conducted using the software Review Manager [59]. For continuous outcomes, we will use standardized mean difference (SMD) with 95% CI, and for dichotomous outcomes, we will use the relative risk (RR) with 95% CI. Calculation and/or transformation of effect sizes into RR or SMD will be done when possible. When effect sizes cannot be calculated or when only one effect size is available for an outcome, we will report the results of this outcome as a narrative synthesis. Due to the anticipated heterogeneity of the data, we will use a random-effects model to pool the effect size of the risk factor for each outcome [60]. Only the adjusted effect sizes will be considered in this model. We will also calculate Higgins’ *I*^2^ statistic, i.e., the percentage of variability in the effect size estimates due to the heterogeneity [61]. The chi-squared test will be used to test the heterogeneity [61, 62]. Moreover, the potential heterogeneity (i.e., *I*^2^ ≥ 50%) will be explored using subgroup analyses based on studies, participants, and exposure characteristics mentioned above.

If sufficient data are available to examine RR and risk factors for long-term opioid therapy among individuals with low socioeconomic status, women, youth, indigenous people, and individuals with a diagnosed mental illness, we will summarize and synthesize the data in a narrative fashion.

### Meta-biases

For clinical trials published after 2005, reporting bias will be assessed by determining whether a protocol for the selected articles was published prior to data collection. We will also assess the publication bias for each outcome by visually examining funnel plots when more than 10 studies are included in the meta-analysis [35]. Finally, we plan to perform the following sensitivity analyses in order to assess the robustness of our results on each outcome of interest: (1) we will explore the individual influence of each study by sequentially removing one at the time from the pooled effect size estimation, (2) we will repeat the pooled effect size estimation by including only studies with low risk of bias, and (3) as we anticipate that confounding variables may vary depending on which studies are included, we will estimate the pooled crude effect size for each outcome using crude effect sizes (non-adjusted) only. Results of these analyses will be compared with the initial pooled effect sizes in order to assess the impact of the confounding variables.

### Confidence in cumulative evidence

For each outcome, we will assess the quality of cumulative evidence with the Grading of Recommendations Assessment, Development and Evaluation (GRADE) [63] to reduce the misinterpretation of the results of the review. This tool is based on five criteria such as the risk of bias in each individual study, the indirectness of the evidence, the heterogeneity of the data, the imprecision of the effect size estimates, and the risk of publication bias. The quality of evidence will be rated high, moderate, low, or very low. As mentioned previously, we will also examine the risk of bias associated with a funding source.

## Discussion

The outcomes of this systematic review will directly inform health care policies and could potentially change the clinical practice of multiple health professionals who are involved in the initial prescriptions of opioids (e.g., anesthesiologists, surgeons, emergency physicians, obstetricians, and physiatrists). The results of this review will modify approach to prolonged and chronic opioid therapy among the providers involved in the renewal and discontinuation of opioid therapy (e.g., pharmacists, primary care physicians, orthopedic surgeons, and psychiatrists), and those practicing at the primary, perioperative and trauma (post-acute treatment decision) care levels. The understanding of the underlying risks for long-term opioid therapy will ultimately contribute to the cost-effect optimization of the patient monitoring processes in the days/weeks following the initiation of opioid therapy. The synthesis data will inform clinical algorithms and help to develop appropriate risk prevention programs.

## Appendix

### Search Strategy

Database: Ovid MEDLINE® in-process and other non-indexed citations, Ovid MEDLINE®, 1946 to present.

**Table:**

*1*	Thoracic Surgery/
*2*	Surgery Department, Hospital/
*3*	Gynecologic Surgical Procedures/
*4*	Ambulatory Surgical Procedures/
*5*	Surgical Procedures, Operative/
*6*	Cardiovascular Surgical Procedures/
*7*	Digestive System Surgical Procedures/
*8*	Elective Surgical Procedures/
*9*	Endocrine Surgical Procedures/
*10*	Minor Surgical Procedures/
*11*	Neurosurgical Procedures/
*12*	Obstetric Surgical Procedures/
*13*	Ophthalmologic Surgical Procedures/
*14*	Orthopedic Procedures/
*15*	Otorhinolaryngologic Surgical Procedures/
*16*	Perioperative Care/
*17*	Reconstructive Surgical Procedures/
*18*	Thoracic Surgical Procedures/
*19*	Cardiovascular System/su [Surgery]
*20*	Bariatric Surgery/
*21*	"Wounds and Injuries"/
*22*	Abdominal Injuries/
*23*	Amputation, Traumatic/
*24*	Arm Injuries/
*25*	Back Injuries/
*26*	BURNS/
*27*	Cold Injury/
*28*	Contrecoup Injury/
*29*	Crush Injuries/
*30*	Electric Injuries/
*31*	Fractures, Bone/
*32*	Hand Injuries/
*33*	Hip Injuries/
*34*	Joint Dislocations/
*35*	Leg Injuries/
*36*	Neck Injuries/
*37*	Occupational Injuries/
*38*	Shoulder Injuries/
*39*	Soft Tissue Injuries/
*40*	"Sprains and Strains"/
*41*	Tendon Injuries/
*42*	Thoracic Injuries/
*43*	Peripheral Nerve Injuries/
*44*	Vascular System Injuries/
*45*	War-Related Injuries/
*46*	Wounds, Nonpenetrating/
*47*	Wounds, Penetrating/
*48*	POSTOPERATIVE CARE/ or POSTOPERATIVE PERIOD/ or Pain, Postoperative/
*49*	(injur* or surger* or surgical or traum* or arthroplast* or accident* or fracture* or dislocation*).tw,sh,kw,kf,oa.
*50*	or/1-49
*51*	exp Analgesics, Opioid/
*52*	exp CODEINE/
*53*	exp MORPHINE/
*54*	CODEINE/
*55*	FENTANYL/
*56*	HYDROCODONE/
*57*	HYDROMORPHONE/
*58*	MEPERIDINE/
*59*	OXYCODONE/
*60*	exp NARCOTICS/
*61*	BUPRENORPHINE/
*62*	BUPRENORPHINE, NALOXONE DRUG COMBINATION/
*63*	METHADONE/
*64*	OXYCODONE/
*65*	OXYMORPHONE/
*66*	PENTAZOCINE/
*67*	TRAMADOL/
*68*	LEVORPHANOL/
*69*	(actiq or adolonta or amadol or analgesic* or anpec or ardinex or asimadolin* or astramorph or avinza or biodalgic or bpethidine or buprenorphine or carfentanil or codeine or codinovo or contramal or demerol or dicodid or dihydrocodeinone or dihydrohydroxycodeinone or dihydromorphinone or dihydrone or dilaudid or dinarkon or dolantin or dolargan or dolcontral or dolosal or dolsin or dur?gesic or dur?morph or epimorph or eucodal or exalgo or fentanest or fentanyl or fentora or fortral or hycodan or hycon or hydrocodeinonebitartrate or hydrocodone or hydrocodone* or hydromorphon or hydromorphone or hydroxyacetanilide or hydroxycodeinon or hysingla or isocodeine or isonipecain or jutadol or kadian or l dromoran or laudacon or levodroman or levodromoran or levo-dromoran or levorphan or levorphanol or lexir or lidol or lorcet or lortab or lydol or meperidine hydrochloride or methadone or morfin or morfine or morphia or morphin or morphine or morphinium or morphium or ms contin or n methylmorphine or narcotic or n-methylmorphine or nobligan or norco or numorphan or operidine or opiate or opioid* or opso or oramorph sr or oripavine or oxecta or oxiconum or oxycodeinon or oxycodone or oxycone or oxycontin or oxymorphone or palladone or pancodine or pentazocine or percocet or pethidine or phentanyl or prontofort or propoxyphene or robidone or roxicet or roxicodone or skenan or sublimaze or takadol or talwin or thebaine or theocodin or theradol or tiral or topalgic or tradol or tradolpuren or tradonal or tralgiol or trama or tramadin or tramadoc or tramadol or tramadolhameln or tramadolor or tramadorsch or tramadura or tramagetic or tramagit or tramake or tramal or tramex or tramundin or trasedal or ultram or vicodin or zamudol or zohydro or zumalgic or zydol or zytram).tw,sh,kw,kf,oa,nm.
*70*	or/51-70
*71*	((chronic* or persistent or long-term or "long term" or prolonged or continu*) adj2 (user* or "use" or usage or pattern or consumption or therap* or effect or effects)).tw,sh,kw,kf,oa.
*72*	PREVALENCE/
*73*	INCIDENCE/
*74*	Risk Assessment/
*75*	Risk Factors/
*76*	("long term" or prolonged or chronic*).tw,sh,kw,kf,oa.
*77*	("risk factors" and ("long term" or prolonged or chronic*)).tw,sh,kw,kf,oa.
*78*	76 and 77
*79*	or/72-75
*80*	78 or 79 or 80
*81*	50 and 71 and 81
*82*	limit 82 to yr="1998 -Current"
*83*	animals/
*84*	humans/
*85*	84 not (84 and 85)
*86*	83 not 86
